# Crystal structure and Hirshfeld surface analysis of hydro­nium 3,5-di­carb­oxy­benzene­sulfonate trihydrate

**DOI:** 10.1107/S2056989026004184

**Published:** 2026-05-07

**Authors:** Kishin Inui, Yukiyasu Kashiwagi, Tomonori Mitsuru

**Affiliations:** a Konishi Chemical Ind. Co. Ltd, 3-4-77 Kozaika, Wakayama 641-0007, Japan; b Osaka Research Institute of Industrial Science and Technology, 1-6-50 Morinomiya Joto-ku Osaka 536-8553 Japan; University of Hyogo, Japan

**Keywords:** crystal structure, hydro­nium ion, hy­dro­gen bond, water cluster, pseudopolymorph

## Abstract

The title com­pound, hydro­nium 3,5-di­carb­oxy­benzene­sulfonate trihydrate, H_3_O^+^·C_8_H_5_O_7_S^−^·3H_2_O, crystallizes in the triclinic space group *P* with one mol­ecule in the asymmetric unit. The structure is the pseudopolymorph with an additional three water mol­ecules to known hydro­nium 3,5-dicarb­oxybenzene­sulfonate. The 3,5-dicarb­oxybenzene­sulfonate (SIP^−^) moiety is surrounded by nine mol­ecules, *i.e.* three SIP^−^ anions, five water mol­ecules and one oxonium ion. The structure, containing a hy­dro­gen-bonded water cluster with eight mol­ecules, forms an *R*_8_^6^(16) ring motif through inter­molecular hy­dro­gen bonding.

## Abstract 2

The title com­pound crystallizes in the triclinic space group *P*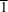. The structure, containing one oxonium ion and a hy­dro­gen-bonded water cluster with eight mol­ecules, forms an *R*_8_^6^(16) ring motif through inter­molecular hy­dro­gen bonding.

## 1. Chemical context

3,5-Di­carb­oxy­benzene­sulfonic acid (also known as 5-sulfo­iso­phthalic acid, SIPA) has a simple structure with two carboxyl groups and one sulfonic acid group on the benzene ring, and has been used in a wide range of fields, especially in industry. As a sulfonated aromatic di­carb­oxy­lic acid, SIPA is a well-known monomer for introducing sulfonic acid into a resin to achieve various functions. For example, SIPA is used as a dyeability modifier for polyesters and polyamides (Ogata *et al.*, 2004 ▸; Vouyiouka *et al.*, 2007 ▸; Oster *et al.*, 2011 ▸; Xiong *et al.*, 2016 ▸), and as monomer of proton-exchange membranes for fuel cells (Bai *et al.*, 2009 ▸). In addition, SIPA is also reported as a raw material for polymer-type ionic liquids used in anti­static agents (Terada, 2009 ▸; Noda, 2010 ▸), thermal acid generators for the manufacture of semiconductor devices (Kaur *et al.*, 2017*a* ▸; Kaur *et al.*, 2017*b* ▸; Kaur *et al.*, 2018 ▸) and dyes for colour filters (Sakamoto *et al.*, 2014 ▸). It is also used in research as a mol­ecular tecton of supra­molecular assemblies and metal–organic frameworks due to its *exo*-trianionic structure. The crystal structure of SIPA was already reported as hydro­nium 3,5-di­carb­oxy­benzenesulfonate (H_3_O^+^·SIP^−^), without additional water molecules (Novozhilova *et al.*, 1989*a* ▸). We have already successfully produced high-purity SIPA that reduced residual sulfuric acid and metal salts on an industrial scale (Inui, 2023 ▸). In an effort to produce high-purity SIPA, we discovered and report here the crystal structure of hydro­nium 3,5-di­carb­oxy­benzene­sulfonate trihydrate (H_3_O^+^·SIP^−^·3H_2_O).

## 2. Structural commentary

**Figure 1:**
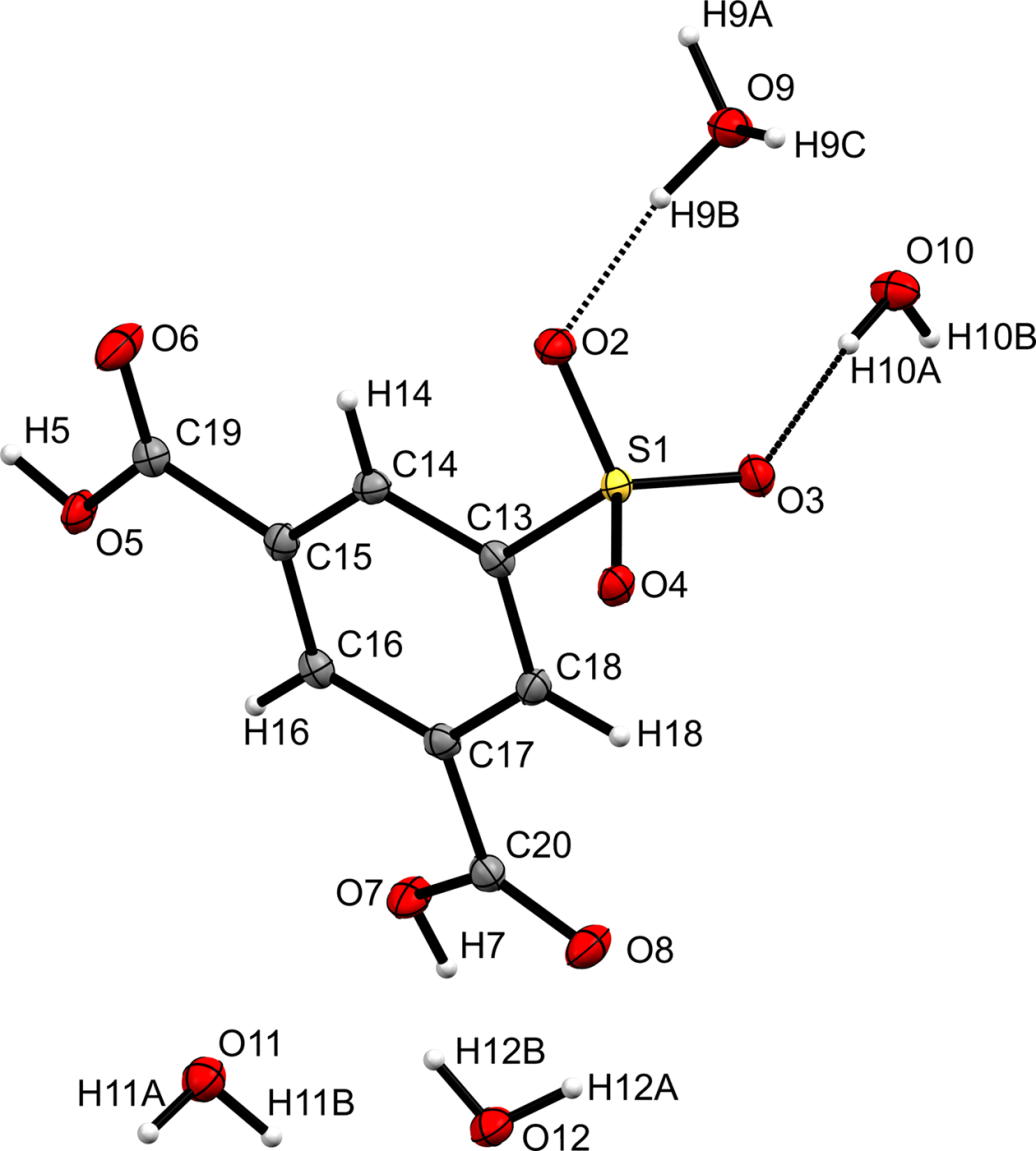
The mol­ecular structure of the title com­pound, with the atom labelling. Displacement ellipsoids are drawn at the 50% probability level. H atoms are repre­sent­ed by spheres of arbitrary radius.

The title com­pound crystallizes in the triclinic space group *P*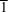 with one mol­ecule in the asymmetric unit (Fig. 1 ▸). The crystal structure is a pseudopolymorph with three additional water
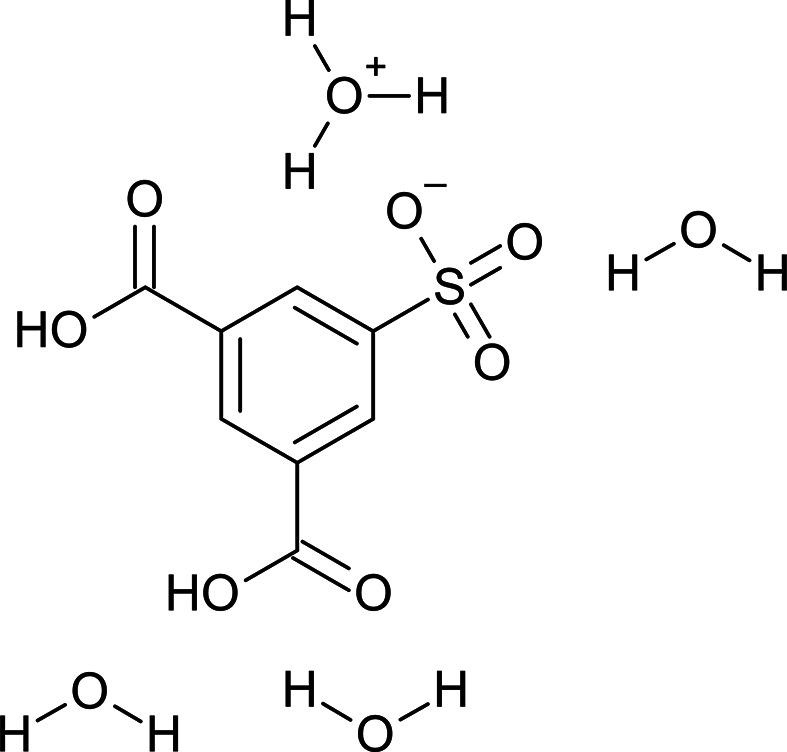
mol­ecules with respect to the known H_3_O^+^·SIP^−^ compound. The arrangement of the two carboxyl groups is mostly planar with respect to benzene ring. The torsion angles C14—C15—C19—O6 and C18—C17—C20—O8 are 3.3 (2) and 11.7 (2)°, respectively.

## 3. Supra­molecular features

**Figure 2:**
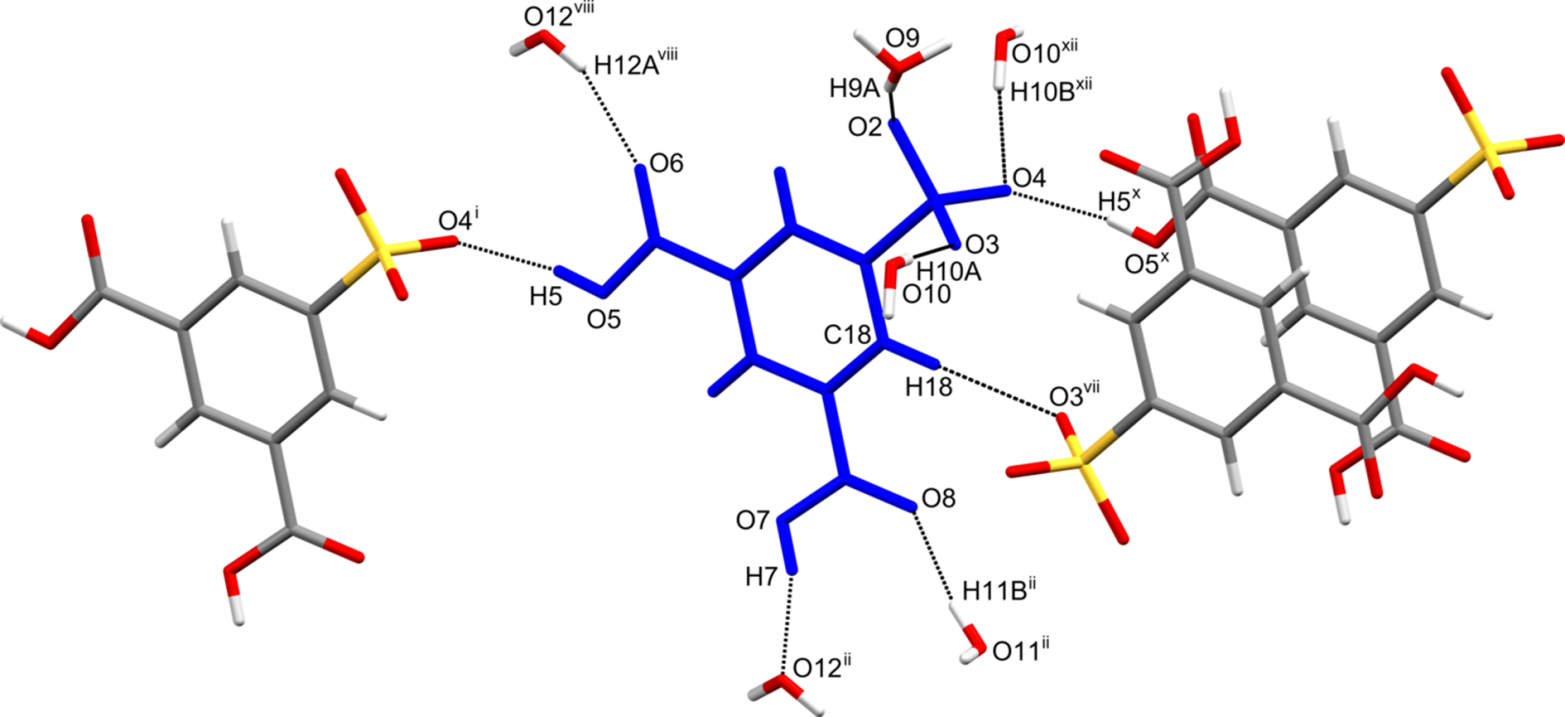
The structure of the SIP^−^ anion (blue) and the surrounding nine mol­ecules. The inter­molecular hy­dro­gen bonds are shown as dashed lines. [Symmetry codes: (i) *x*, *y* + 1, *z*; (ii) −*x* + 1, −*y* + 2, −*z*; (vii) −*x* + 1, −*y* + 1, −*z* + 1; (viii) −*x*, −*y* + 2, −*z* + 1; (*x*) *x*, *y* − 1, *z*; (xii) *x* − 1, *y*, *z*.]

**Figure 3:**
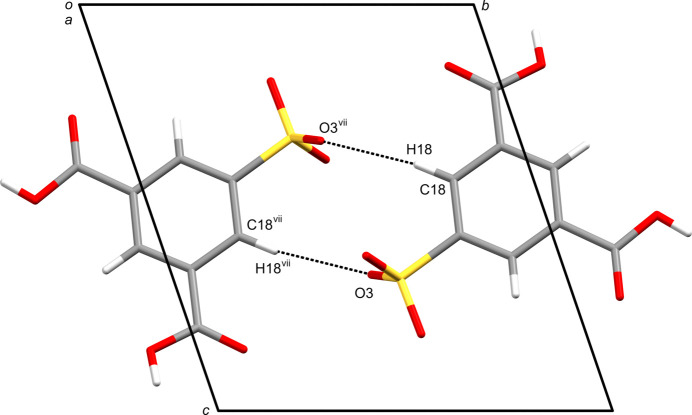
The centrosymmetric dimeric structure of the H_3_O^+^·SIP^−^·3H_2_O. The inter­molecular C18—H18⋯O3 hy­dro­gen bonds are shown as dashed lines. Solvated water mol­ecules have been omitted for clarity. [Symmetry code: (vii) −*x* + 1, −*y* + 1, −*z* + 1.]

**Figure 4:**
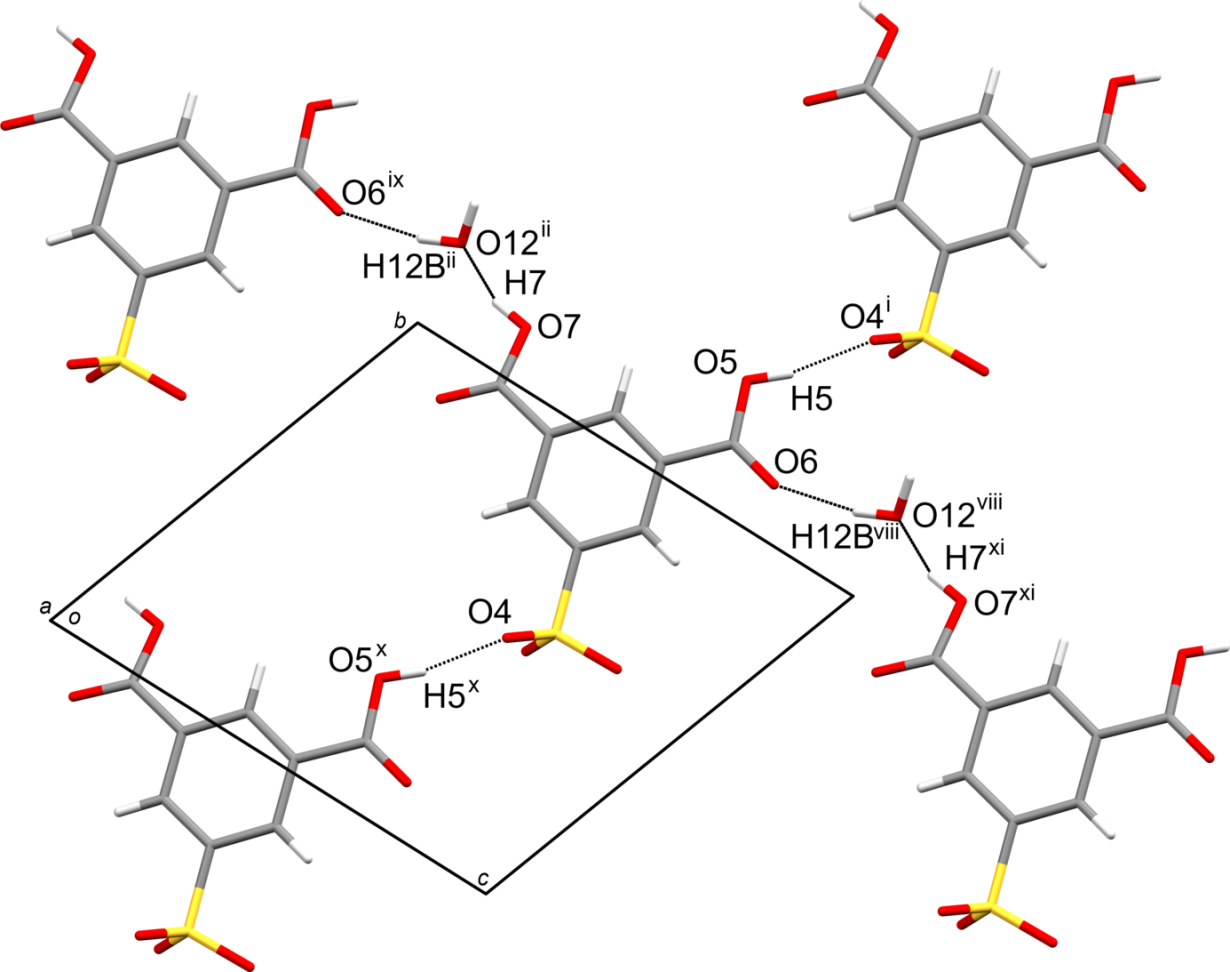
Two-dimensional sheet structure between SIP^−^ anions and water mol­ecules parallel to (101). The inter­molecular O5—H5⋯O4^i^, O5^x^—H5^x^⋯O4, O7—H7⋯O12^ii^, O12^ii^—H12B^ii^⋯O6^ix^, O7^xi^—H7^xi^⋯O12^viii^ and O12^viii^—H12B^viii^⋯O6 hy­dro­gen bonds are shown as dashed lines. Water mol­ecules not involved in the inter­actions have been omitted for clarity. [Symmetry codes: (i) *x*, *y* + 1, *z*; (ii) −*x* + 1, −*y* + 2, −*z*; (viii) −*x*, −*y* + 2, −*z* + 1; (ix) *x* + 1, *y*, *z* − 1; (x) *x*, *y* − 1, *z*; (xi) *x* − 1, *y*, *z* + 1.]

**Figure 5:**
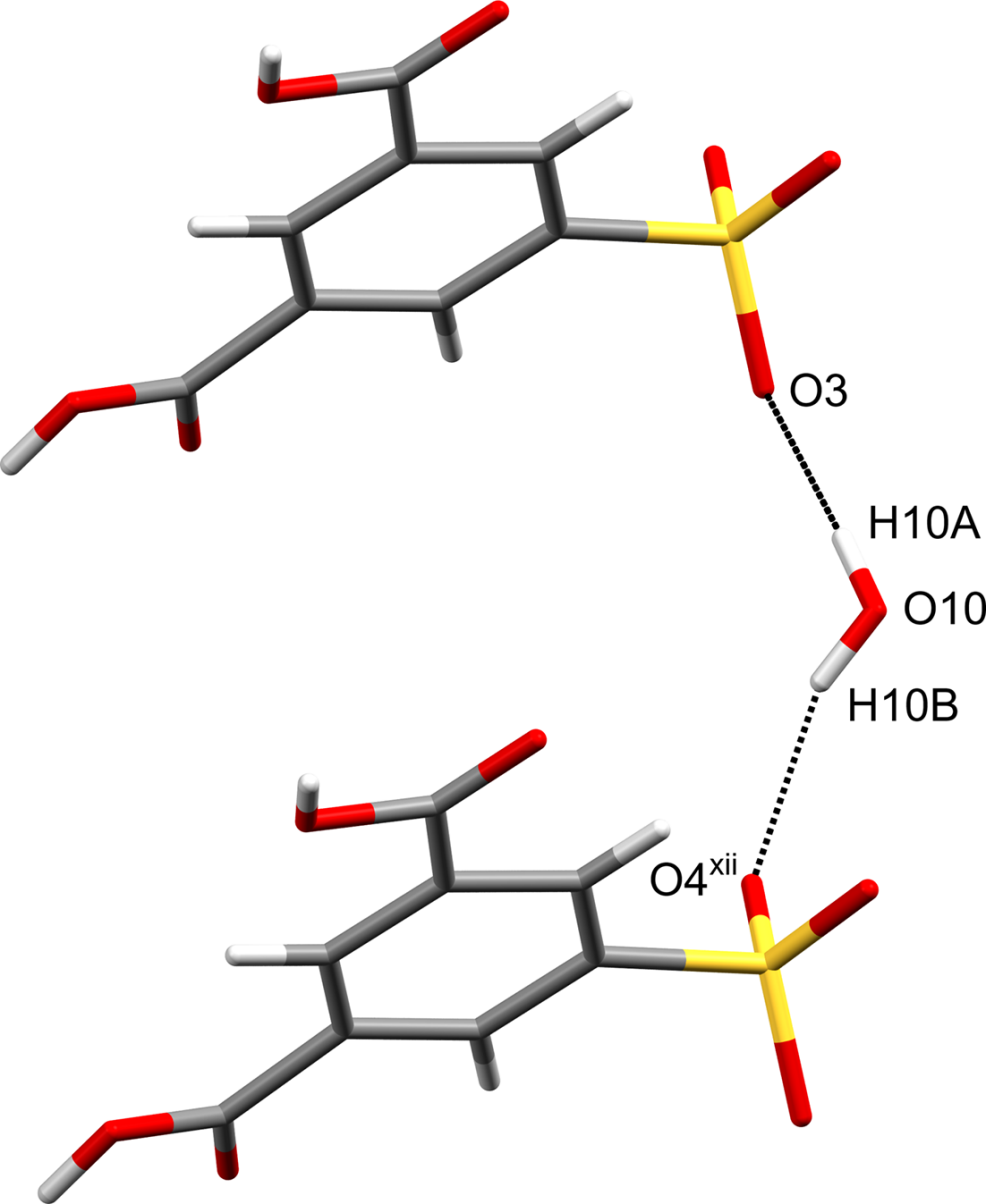
One-dimensional chain structure between SIP^−^ anions and water mol­ecules along the *a* axis. The inter­molecular O10—H10*A*⋯O3 and O10—H10*B*⋯O4^xii^ hy­dro­gen bonds are shown as dashed lines. Water mol­ecules not involved in the inter­actions have been omitted for clarity. [Symmetry code: (xii) *x* − 1, *y*, *z*.]

**Figure 6:**
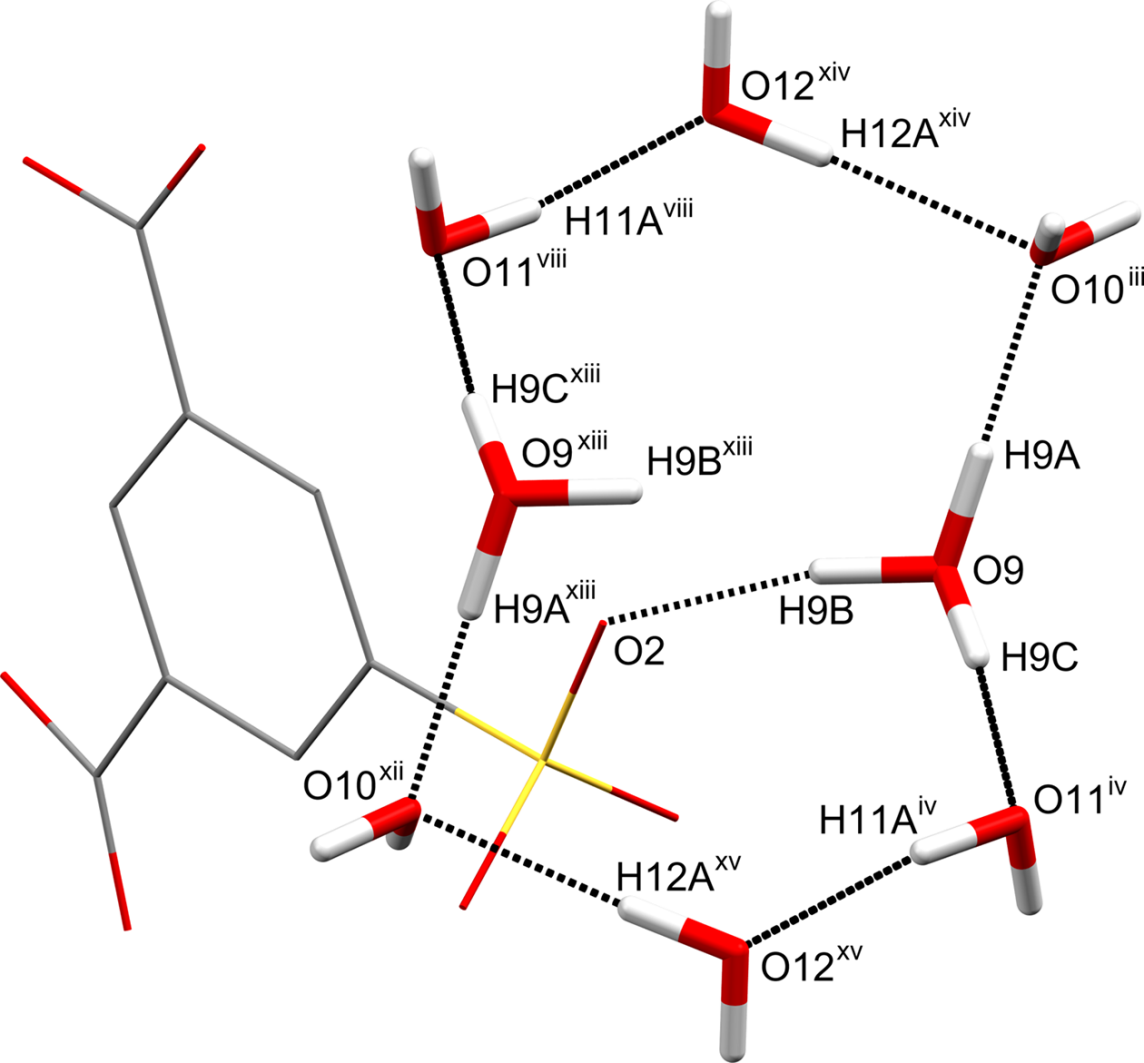
The 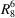
(16) ring motif formed by inter­molecular O—H⋯O hy­dro­gen bonds involving eight water mol­ecules. The inter­molecular O—H⋯O hy­dro­gen bonds are shown as dashed lines. The SIP^−^ anions in the asymmetric unit are shown in wireframe style and the H atoms have been omitted. The inter­molecular O9—H9*B*⋯O2 hy­dro­gen bond is also shown as a dashed line. [Symmetry codes: (iii) −*x* + 1, −*y* + 1, −*z* + 2; (iv) *x*, *y* − 1, *z* + 1; (viii) −*x*, −*y* + 2, −*z* + 1; (xii) *x* − 1, *y*, *z*; (xiii) −*x*, −*y* + 1, −*z* + 2; (xiv) *x*, *y*, *z* + 1; (xv) −*x*, −*y* + 1, −*z* + 1.]

The 3,5-di­carb­oxy­benzene­sulfonate (SIP^−^) moiety is sur­rounded by nine mol­ecules, three SIP^−^ anions, five water mol­ecules and one hydro­nium ion, involved in nine hy­dro­gen bonds of eight different kinds (Table 1 ▸ and Fig. 2 ▸). In the crystal, SIP^−^ anions are linked by inter­molecular C—H⋯O hy­dro­gen bonds [C18—H18⋯O3^vii^; symmetry code: (vii) −*x* + 1, −*y* + 1, −*z* + 1], forming an inversion dimer with 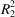
(10) ring motifs (Fig. 3 ▸). The sheet structure of SIP^−^ anions and water mol­ecules is formed by inter­molecular hy­dro­gen-bond networks parallel to (101), as shown in Fig. 4 ▸. One one-dimensional chain structure is formed by O—H⋯O hy­dro­gen bonds [O5—H5⋯O4^i^ and O5^x^—H5^x^⋯O4; symmetry codes: (i) *x*, *y* + 1, *z*; (x) *x*, *y* − 1, *z*] and another one-dimensional chain structure is formed by two kinds of O—H⋯O hy­dro­gen bonds [O7—H7⋯O12^ii^, O12^ii^—H12*B*^ii^⋯O6^ix^, O7^xi^—H7^xi^⋯O12^viii^ and O12^viii^—H12*B*^viii^⋯O6; symmetry codes: (ii) −*x* + 1, −*y* + 2, −*z*; (viii) −*x*, −*y* + 2, −*z* + 1; (ix) *x* + 1, *y*, *z* − 1; (xi) *x* − 1, *y*, *z* + 1] due to an inter­mediate water mol­ecule. Fig. 5 ▸ shows the one-dimensional chain structure formed by two inter­molecular O—H⋯O hy­dro­gen bonds [O10—H10*A*⋯O3 and O10—H10*B*⋯O4^xii^; symmetry code: (xii) *x* − 1, *y*, *z*] between a water mol­ecule and SIP^−^ anions along the *a* axis. In the crystal, the SIP^−^ anions and water mol­ecules containing atoms O10 and O12 are linked by inter­molecular O—H⋯O hy­dro­gen bonds, forming a three-dimensional network structure. Furthermore, focusing on the water mol­ecules and the hydro­nium ion, the structure containing a hy­dro­gen-bonded water cluster with eight mol­ecules forms an 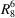
(16) ring motif through inter­molecular O—H⋯O hy­dro­gen bonding [O9—H9*A*⋯O10^iii^, O9—H9*C*⋯O11^iv^, O11^iv^—H11*A*^iv^⋯O12^xv^, O12^xv^—H12*A*^xv^⋯O10^xii^, O9^xiii^—H9*A*^xiii^⋯O10^xii^, O9^xiii^—H9*C*^xiii^⋯O11^viii^, O11^viii^—O11*A*^viii^⋯O12^xiv^ and O12^xiv^—O12*A*^xiv^⋯O10^iii^; symmetry codes: (iii) −*x* + 1, −*y* + 1, −*z* + 2; (iv) *x*, *y* − 1, *z* + 1; (viii) −*x*, −*y* + 2, −*z* + 1; (xii) *x* − 1, *y*, *z*; (xiii) −*x*, −*y* + 1, −*z* + 2; (xiv) *x*, *y*, *z* + 1; (xv) −*x*, −*y* + 1, −*z* + 1] (Fig. 6 ▸). The water cluster is surrounded by eight SIP^−^ anions and the water clusters do not interact with each other.

**Figure 7:**
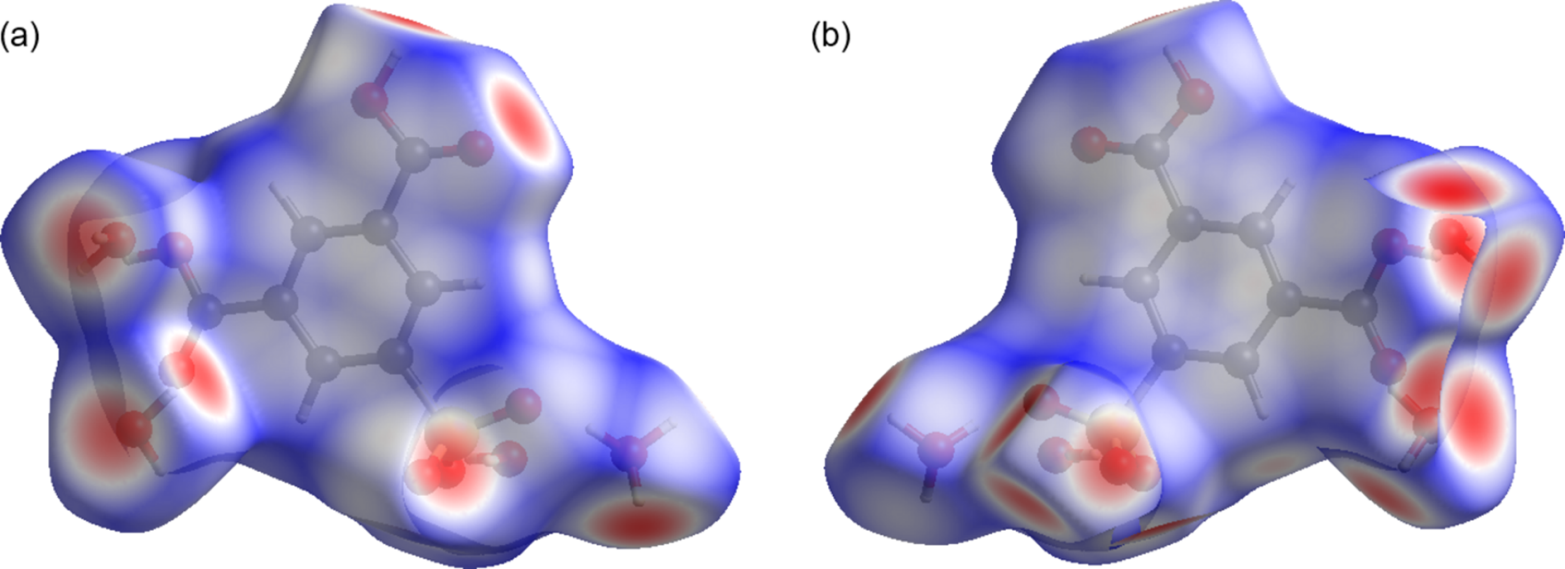
Hirshfeld surface mapped over *d*_norm_ for the title com­pound, showing (*a*) a front view and (*b*) a back view. Red spots indicate short O—H⋯O contacts.

**Figure 8:**
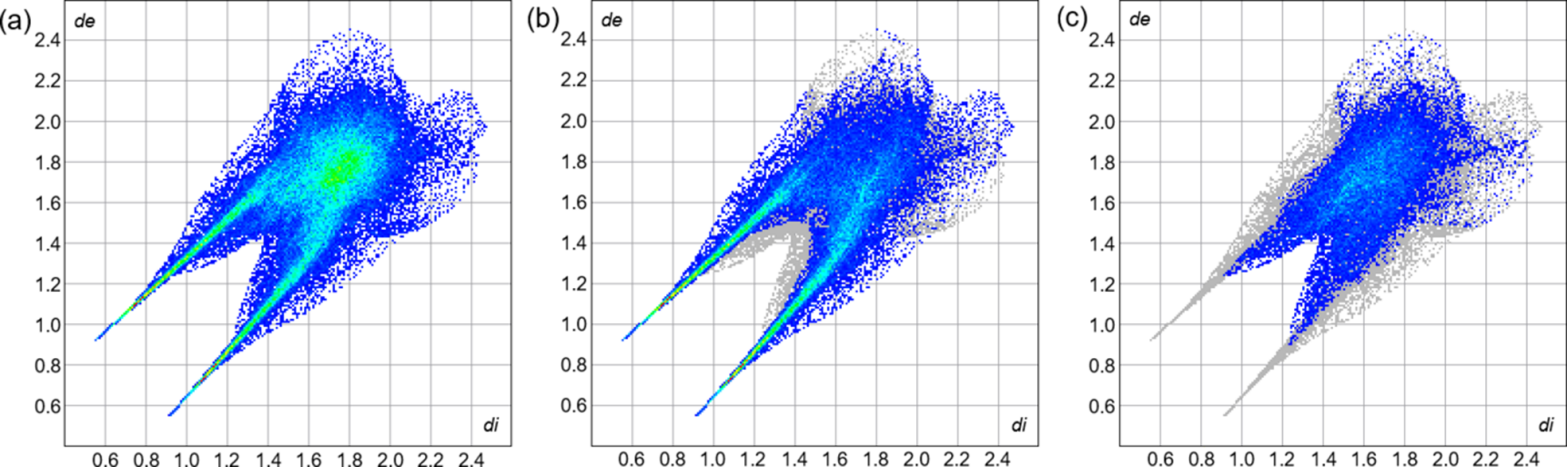
Two-dimensional fingerprint plots for the title com­pound. (*a*) Full fingerprint plot showing the overall distribution of *d*_i_ and *d*_e_. Fingerprint plots highlighting the (*b*) O⋯H/H⋯O contacts and (*c*) H⋯H contacts.

To visualize the inter­molecular inter­actions in the crystal of the title com­pound, a Hirshfeld surface (HS) analysis (Spackman & Jayatilaka, 2009 ▸) was carried out using *CrystalExplorer* (Version 21.5; Spackman *et al.*, 2021 ▸). The HS mapped over *d*_norm_ shows several red spots, which mostly correspond to short O—H⋯O contacts between neighbouring mol­ecules (Fig. 7 ▸). The percentage contributions of the inter­molecular inter­actions to the total HS were qu­anti­fied by two-dimensional fingerprint plots (McKinnon *et al.*, 2007 ▸). The fingerprint plots of *d*_i_*versus d*_e_ shown in Fig. 8 ▸ reveal that the most significant contributions arise from O⋯H/H⋯O (55.9%) and H⋯H (26.4%) contacts. Smaller contributions are observed for C⋯C (6.4%), O⋯O (4.2%), O⋯C/C⋯O (4.0%) and C⋯H/H⋯C (3.1%) inter­actions.

## 4. Database survey

A search of the Cambridge Structural Database (CSD, Version 6.00, update August 2025; Groom *et al.*, 2016 ▸) using *ConQuest* (Bruno *et al.*, 2002 ▸) for com­pounds containing the the 1-sulfonato-3,5-di­carboxyl­ato­benzene skeleton gave 151 hits with combinations of localized carboxyl­ate and localized sulfonate. There are four combinations of notations, two kinds of carboxyl­ates (localized: two C=O double bonds and two C—O single bond; delocalized: four delocalized carbon–oxygen bonds) and two kinds of sulfonates (localized: two S=O double bonds and one S—O single bond; delocalized: one S=O double bond and two delocalized S—O bonds). The survey for the combination of localized/delocalized carboxyl­ates/sulfonate gave 151 hits of localized carboxyl­ates and localized sulfonate (see above), 218 hits of delocalized carboxyl­ates and localized sulfonate, two hits of localized carboxyl­ates and delocalized sulfonate, and 31 hits of delocalized carboxyl­ates and delocalized sulfonate. To refine the search for ‘organic’ structures gave 15 hits from only a combination of delocalized carboxyl­ates and delocalized sulfonate. The carboxyl­ates are protonated in 14 structures and only the crystal structure of the potassium salt of SIPA is partially deprotonated (CSD refcode KIBJUA; Novozhilova *et al.*, 1989*b* ▸). All of 15 structures contain anionic sulfonate structures and the counter-cations are six hits of protonated pyridinium, one hit of methyl pyridinium, four hits of diprotonated secondary ammonium, two hits of protonated imidazolium, one hit of potassium and one hit of oxonium. The oxonium com­pound was reported previously, *i.e.* H_3_O^+^·SIP^−^ (JEJLOY; Novozhilova *et al.*, 1989*a* ▸).

## 5. Synthesis and crystallization

H_3_O^+^·SIP^−^ (Konishi Chemical Ind. Co. Ltd, 163 g, 0.59 mol) was suspended in water (61 ml) and stirred at room temperature for 3 h. During stirring, the crystal was transformed from H_3_O^+^·SIP^−^ to H_3_O^+^·SIP^−^·3H_2_O due to solvation. After that, the suspension was filtered off and the solids were collected as the seed crystals of H_3_O^+^·SIP^−^·3H_2_O (73 g, 0.23 mol). To prepare the supersaturated solution of SIPA, a suspension of H_3_O^+^·SIP^−^ (0.25 *M*, 143 ml) was com­pletely dissolved at 329 K and then cooled to room temperature. A small amount of the seed crystals of H_3_O^+^·SIP^−^·3H_2_O was added to the supersaturated solution of SIPA, which was then sealed to prevent dehydration. After storing at room temperature for several days, colourless crystals suitable for X-ray analysis were obtained.

## 6. Refinement

Crystal data, data collection and structure refinement details are summarized in Table 2 ▸. C-bound H atoms were placed in geometrically calculated positions (C—H = 0.95 Å) and refined as part of a riding model with *U*_iso_(H) = 1.2*U*_eq_(C). The O-bound H atoms H5, H7, H9*A*, H9*B*, H10*A*, H10*B*, H11*A*, H11*B*, H12*A* and H12*B* were located in a difference Fourier map and refined freely. Atom H9*C* was located in the difference Fourier map but was refined with a distance restraint of O—H = 0.84 ± 0.02 Å.

## Supplementary Material

Crystal structure: contains datablock(s) I. DOI: 10.1107/S2056989026004184/ox2020sup1.cif

Structure factors: contains datablock(s) I. DOI: 10.1107/S2056989026004184/ox2020Isup2.hkl

CCDC reference: 2548048

Additional supporting information:  crystallographic information; 3D view; checkCIF report

## Figures and Tables

**Table 1:** Hydrogen-bond geometry (Å, °)

*D*—H⋯*A*	*D*—H	H⋯*A*	*D*⋯*A*	*D*—H⋯*A*
O5—H5⋯O4^i^	0.86 (3)	1.84 (3)	2.6850 (15)	168 (2)
O7—H7⋯O12^ii^	0.87 (2)	1.79 (2)	2.6236 (16)	160 (3)
O9—H9*A*⋯O10^iii^	0.94 (3)	1.64 (3)	2.5811 (16)	175 (2)
O9—H9*B*⋯O2	0.93 (2)	1.70 (3)	2.6124 (15)	168 (3)
O9—H9*C*⋯O11^iv^	0.91 (2)	1.54 (2)	2.4469 (17)	173 (3)
O10—H10*A*⋯O3	0.83 (2)	1.89 (2)	2.7167 (16)	175 (2)
O10—H10*B*⋯O4^v^	0.83 (3)	1.98 (3)	2.7778 (16)	161 (3)
O11—H11*A*⋯O12^vi^	0.89 (3)	1.89 (3)	2.7708 (17)	173 (3)
O11—H11*B*⋯O8^ii^	0.86 (3)	1.84 (3)	2.6834 (16)	168 (3)
O12—H12*A*⋯O10^vii^	0.87 (3)	1.90 (3)	2.7604 (17)	174 (2)
O12—H12*B*⋯O6^viii^	0.83 (3)	1.94 (3)	2.7373 (17)	161 (3)
C18—H18⋯O3^vii^	0.95	2.57	3.5008 (19)	167

Symmetry codes: (i) 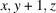; (ii) 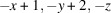; (iii) 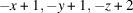; (iv) 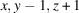; (v) 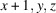; (vi) 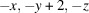; (vii) 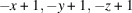; (viii) 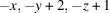.

**Table 2:** Experimental details

Crystal data	
Chemical formula	H_3_O^+^·C_8_H_5_O_7_S^−^·3H_2_O
*M*_r_	318.25
Crystal system, space group	Triclinic, *P* 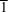
Temperature (K)	100
*a*, *b*, *c* (Å)	7.0692 (2), 9.4888 (2), 10.5484 (2)
α, β, γ (°)	70.482 (2), 77.103 (2), 85.021 (2)
*V* (Å^3^)	650.03 (3)
*Z*	2
Radiation type	Cu *K*α
μ (mm^−1^)	2.78
Crystal size (mm)	0.29 × 0.08 × 0.03

Data collection	
Diffractometer	Rigaku XtaLAB Synergy Dualflex HyPix
Absorption correction	Multi-scan (*CrysAlis PRO*; Rigaku OD, 2023 ▸)
*T*_min_, *T*_max_	0.682, 1.000
No. of measured, independent and observed [*I* > 2σ(*I*)] reflections	7050, 2534, 2395
*R*_int_	0.026
(sin θ/λ)_max_ (Å^−1^)	0.632

Refinement	
*R*[*F*^2^ > 2σ(*F*^2^)], *wR*(*F*^2^), *S*	0.029, 0.078, 1.08
No. of reflections	2534
No. of parameters	225
No. of restraints	1
H-atom treatment	H atoms treated by a mixture of independent and constrained refinement
Δρ_max_, Δρ_min_ (e Å^−3^)	0.31, −0.55

Computer programs: *CrysAlis PRO*; Rigaku OD, 2023 ▸), *SHELXT2018* (Sheldrick, 2015*a* ▸), *SHELXL2018* (Sheldrick, 2015*b* ▸) and *OLEX2* (Dolomanov *et al.*, 2009 ▸).

## supplementary crystallographic information

### Hydronium 3,5-dicarboxybenzenesulfonate trihydrate Crystal data

**Table:**

H_3_O^+^·C_8_H_5_O_7_S^−^·3H_2_O	*Z* = 2
*M**_r_* = 318.25	*F*(000) = 332
Triclinic, *P*1	*D*_x_ = 1.626 Mg m^−^^3^
*a* = 7.0692 (2) Å	Cu *K*α radiation, λ = 1.54184 Å
*b* = 9.4888 (2) Å	Cell parameters from 4896 reflections
*c* = 10.5484 (2) Å	θ = 4.5–76.6°
α = 70.482 (2)°	µ = 2.78 mm^−^^1^
β = 77.103 (2)°	*T* = 100 K
γ = 85.021 (2)°	Block, colourless
*V* = 650.03 (3) Å^3^	0.29 × 0.08 × 0.03 mm

### Hydronium 3,5-dicarboxybenzenesulfonate trihydrate Data collection

**Table:**

Rigaku XtaLAB Synergy Dualflex HyPix diffractometer	2534 independent reflections
Radiation source: micro-focus sealed X-ray tube, PhotonJet (Cu) X-ray Source	2395 reflections with *I* > 2σ(*I*)
Mirror monochromator	*R*_int_ = 0.026
Detector resolution: 10.0000 pixels mm^-1^	θ_max_ = 76.9°, θ_min_ = 4.6°
ω scans	*h* = −5→8
Absorption correction: multi-scan (*CrysAlis PRO*; Rigaku OD, 2023)	*k* = −11→11
*T*_min_ = 0.682, *T*_max_ = 1.000	*l* = −12→12
7050 measured reflections	

### Hydronium 3,5-dicarboxybenzenesulfonate trihydrate Refinement

**Table:**

Refinement on *F*^2^	Primary atom site location: dual
Least-squares matrix: full	Hydrogen site location: mixed
*R*[*F*^2^ > 2σ(*F*^2^)] = 0.029	H atoms treated by a mixture of independent and constrained refinement
*wR*(*F*^2^) = 0.078	*w* = 1/[σ^2^(*F*_o_^2^) + (0.0409*P*)^2^ + 0.2593*P*] where *P* = (*F*_o_^2^ + 2*F*_c_^2^)/3
*S* = 1.08	(Δ/σ)_max_ < 0.001
2534 reflections	Δρ_max_ = 0.31 e Å^−^^3^
225 parameters	Δρ_min_ = −0.55 e Å^−^^3^
1 restraint	

### Hydronium 3,5-dicarboxybenzenesulfonate trihydrate Special details

**Table:**

Geometry. All esds (except the esd in the dihedral angle between two l.s. planes) are estimated using the full covariance matrix. The cell esds are taken into account individually in the estimation of esds in distances, angles and torsion angles; correlations between esds in cell parameters are only used when they are defined by crystal symmetry. An approximate (isotropic) treatment of cell esds is used for estimating esds involving l.s. planes.

### Hydronium 3,5-dicarboxybenzenesulfonate trihydrate Fractional atomic coordinates and isotropic or equivalent isotropic displacement parameters (Å^2^)

**Table:**

	*x*	*y*	*z*	*U*_iso_*/*U*_eq_	
S1	0.22880 (5)	0.57553 (3)	0.67370 (3)	0.01079 (11)	
O2	0.13855 (15)	0.58112 (11)	0.81082 (10)	0.0159 (2)	
O3	0.42016 (14)	0.50675 (11)	0.66474 (11)	0.0156 (2)	
O4	0.09963 (14)	0.50963 (11)	0.61847 (10)	0.0137 (2)	
O5	0.19654 (15)	1.28201 (11)	0.51791 (11)	0.0158 (2)	
H5	0.164 (3)	1.345 (3)	0.562 (2)	0.034 (6)*	
O6	0.05838 (17)	1.11710 (12)	0.71919 (11)	0.0237 (3)	
O7	0.55512 (16)	1.12016 (11)	0.14810 (11)	0.0171 (2)	
H7	0.637 (3)	1.132 (3)	0.070 (3)	0.039 (6)*	
O8	0.54887 (16)	0.88310 (12)	0.15135 (11)	0.0198 (2)	
C13	0.25703 (19)	0.76498 (15)	0.56670 (15)	0.0112 (3)	
C14	0.18601 (19)	0.87815 (16)	0.62074 (14)	0.0121 (3)	
H14	0.116744	0.854872	0.713265	0.015*	
C15	0.21747 (19)	1.02717 (15)	0.53748 (15)	0.0121 (3)	
C16	0.3168 (2)	1.06126 (15)	0.40202 (15)	0.0123 (3)	
H16	0.338316	1.162595	0.346005	0.015*	
C17	0.38506 (19)	0.94608 (16)	0.34825 (15)	0.0124 (3)	
C18	0.3562 (2)	0.79706 (16)	0.43054 (15)	0.0126 (3)	
H18	0.403445	0.718560	0.394306	0.015*	
C19	0.1473 (2)	1.14599 (16)	0.60156 (15)	0.0130 (3)	
C20	0.5028 (2)	0.97893 (16)	0.20580 (15)	0.0134 (3)	
O9	0.30252 (15)	0.41647 (12)	1.01119 (12)	0.0174 (2)	
H9A	0.285 (3)	0.448 (3)	1.089 (3)	0.041 (6)*	
H9B	0.247 (3)	0.486 (3)	0.944 (3)	0.043 (6)*	
H9C	0.251 (4)	0.326 (2)	1.028 (3)	0.073 (9)*	
O12	0.16413 (16)	0.79716 (13)	0.07030 (12)	0.0180 (2)	
H12A	0.206 (3)	0.710 (3)	0.115 (3)	0.041 (6)*	
H12B	0.118 (4)	0.836 (3)	0.130 (3)	0.050 (7)*	
O11	0.16454 (17)	1.17783 (12)	0.03829 (12)	0.0188 (2)	
H11A	0.057 (4)	1.193 (3)	0.005 (2)	0.037 (6)*	
H11B	0.247 (4)	1.147 (3)	−0.020 (3)	0.051 (7)*	
O10	0.72986 (17)	0.48737 (12)	0.78303 (11)	0.0175 (2)	
H10A	0.631 (3)	0.496 (2)	0.750 (2)	0.031 (6)*	
H10B	0.828 (4)	0.510 (3)	0.721 (3)	0.054 (8)*	

### Hydronium 3,5-dicarboxybenzenesulfonate trihydrate Atomic displacement parameters (Å^2^)

**Table:**

	*U*^11^	*U*^22^	*U*^33^	*U*^12^	*U*^13^	*U*^23^
S1	0.01341 (18)	0.00786 (17)	0.01068 (18)	0.00055 (12)	−0.00252 (13)	−0.00262 (13)
O2	0.0221 (5)	0.0127 (5)	0.0113 (5)	0.0005 (4)	−0.0021 (4)	−0.0030 (4)
O3	0.0157 (5)	0.0137 (5)	0.0172 (5)	0.0026 (4)	−0.0054 (4)	−0.0042 (4)
O4	0.0159 (5)	0.0103 (5)	0.0163 (5)	−0.0004 (4)	−0.0040 (4)	−0.0057 (4)
O5	0.0222 (5)	0.0085 (5)	0.0159 (5)	0.0017 (4)	−0.0015 (4)	−0.0050 (4)
O6	0.0359 (6)	0.0151 (5)	0.0165 (6)	−0.0013 (5)	0.0059 (5)	−0.0073 (4)
O7	0.0215 (5)	0.0134 (5)	0.0123 (5)	−0.0020 (4)	0.0032 (4)	−0.0028 (4)
O8	0.0259 (6)	0.0165 (5)	0.0156 (5)	−0.0011 (4)	0.0023 (4)	−0.0075 (4)
C13	0.0115 (6)	0.0098 (7)	0.0125 (7)	−0.0004 (5)	−0.0044 (5)	−0.0025 (5)
C14	0.0122 (6)	0.0135 (7)	0.0104 (7)	−0.0004 (5)	−0.0019 (5)	−0.0039 (5)
C15	0.0109 (6)	0.0123 (7)	0.0142 (7)	0.0005 (5)	−0.0036 (5)	−0.0054 (6)
C16	0.0118 (6)	0.0106 (7)	0.0139 (7)	−0.0001 (5)	−0.0037 (5)	−0.0026 (5)
C17	0.0116 (6)	0.0140 (7)	0.0120 (7)	0.0000 (5)	−0.0036 (5)	−0.0043 (6)
C18	0.0130 (6)	0.0125 (7)	0.0139 (7)	0.0016 (5)	−0.0034 (5)	−0.0064 (5)
C19	0.0128 (6)	0.0120 (7)	0.0140 (7)	0.0004 (5)	−0.0032 (5)	−0.0038 (6)
C20	0.0132 (6)	0.0132 (7)	0.0137 (7)	0.0012 (5)	−0.0043 (5)	−0.0036 (6)
O9	0.0215 (5)	0.0167 (5)	0.0139 (5)	−0.0001 (4)	−0.0049 (4)	−0.0042 (4)
O12	0.0206 (5)	0.0188 (6)	0.0137 (5)	0.0015 (4)	−0.0002 (4)	−0.0066 (5)
O11	0.0186 (6)	0.0196 (6)	0.0176 (6)	0.0004 (4)	−0.0012 (5)	−0.0072 (5)
O10	0.0142 (5)	0.0235 (6)	0.0140 (6)	−0.0023 (4)	−0.0025 (5)	−0.0051 (4)

### Hydronium 3,5-dicarboxybenzenesulfonate trihydrate Geometric parameters (Å, º)

**Table:**

S1—O2	1.4614 (10)	C13—C14	1.3849 (19)
S1—O3	1.4477 (10)	C13—C18	1.394 (2)
S1—O4	1.4643 (10)	C14—C15	1.399 (2)
S1—C13	1.7752 (14)	C15—C16	1.386 (2)
O5—C19	1.3212 (18)	C15—C19	1.4956 (19)
O6—C19	1.2100 (18)	C16—C17	1.396 (2)
O7—C20	1.3205 (18)	C17—C18	1.395 (2)
O8—C20	1.2153 (18)	C17—C20	1.490 (2)
			
O2—S1—O4	111.58 (6)	C16—C15—C19	121.91 (13)
O2—S1—C13	105.41 (6)	C15—C16—C17	119.75 (13)
O3—S1—O2	113.95 (6)	C16—C17—C20	120.93 (13)
O3—S1—O4	112.28 (6)	C18—C17—C16	120.38 (13)
O3—S1—C13	107.03 (6)	C18—C17—C20	118.52 (12)
O4—S1—C13	105.90 (6)	C13—C18—C17	119.04 (13)
C14—C13—S1	119.65 (11)	O5—C19—C15	113.05 (12)
C14—C13—C18	121.17 (13)	O6—C19—O5	124.70 (13)
C18—C13—S1	119.14 (10)	O6—C19—C15	122.23 (13)
C13—C14—C15	119.23 (13)	O7—C20—C17	113.15 (12)
C14—C15—C19	117.63 (13)	O8—C20—O7	124.28 (13)
C16—C15—C14	120.42 (13)	O8—C20—C17	122.54 (13)

### Hydronium 3,5-dicarboxybenzenesulfonate trihydrate Hydrogen-bond geometry (Å, º)

**Table:**

*D*—H···*A*	*D*—H	H···*A*	*D*···*A*	*D*—H···*A*
O5—H5···O4^i^	0.86 (3)	1.84 (3)	2.6850 (15)	168 (2)
O7—H7···O12^ii^	0.87 (2)	1.79 (2)	2.6236 (16)	160 (3)
O9—H9*A*···O10^iii^	0.94 (3)	1.64 (3)	2.5811 (16)	175 (2)
O9—H9*B*···O2	0.93 (2)	1.70 (3)	2.6124 (15)	168 (3)
O9—H9*C*···O11^iv^	0.91 (2)	1.54 (2)	2.4469 (17)	173 (3)
O10—H10*A*···O3	0.83 (2)	1.89 (2)	2.7167 (16)	175 (2)
O10—H10*B*···O4^v^	0.83 (3)	1.98 (3)	2.7778 (16)	161 (3)
O11—H11*A*···O12^vi^	0.89 (3)	1.89 (3)	2.7708 (17)	173 (3)
O11—H11*B*···O8^ii^	0.86 (3)	1.84 (3)	2.6834 (16)	168 (3)
O12—H12*A*···O10^vii^	0.87 (3)	1.90 (3)	2.7604 (17)	174 (2)
O12—H12*B*···O6^viii^	0.83 (3)	1.94 (3)	2.7373 (17)	161 (3)
C18—H18···O3^vii^	0.95	2.57	3.5008 (19)	167

Symmetry codes: (i) *x*, *y*+1, *z*; (ii) −*x*+1, −*y*+2, −*z*; (iii) −*x*+1, −*y*+1, −*z*+2; (iv) *x*, *y*−1, *z*+1; (v) *x*+1, *y*, *z*; (vi) −*x*, −*y*+2, −*z*; (vii) −*x*+1, −*y*+1, −*z*+1; (viii) −*x*, −*y*+2, −*z*+1.

## References

[bb1] Bai, H. & Ho, W. S. W. (2009). *J. Taiwan Inst. Chem. Eng.* **40**, 260–267.

[bb2] Bruno, I. J., Cole, J. C., Edgington, P. R., Kessler, M., Macrae, C. F., McCabe, P., Pearson, J. & Taylor, R. (2002). *Acta Cryst.* B**58**, 389–397. 10.1107/s0108768102003324 12037360

[bb3] Dolomanov, O. V., Bourhis, L. J., Gildea, R. J., Howard, J. A. K. & Puschmann, H. (2009). *J. Appl. Cryst.* **42**, 339–341.

[bb4] Groom, C. R., Bruno, I. J., Lightfoot, M. P. & Ward, S. C. (2016). *Acta Cryst.* B**72**, 171–179. 10.1107/S2052520616003954 PMC4822653 27048719

[bb5] Inui, K. (2023). Jpn Patent 2023043678.

[bb6] Kaur, I., Kang, D., Liu, C., Pohlers, G. & Li, M. (2018). US Patent 20180118968.

[bb7] Kaur, I., Liu, C., Rowell, K., Pohlers, G. & Li, M. (2017*a*). US Patent 20170123313.

[bb8] Kaur, I., Liu, C., Rowell, K., Pohlers, G. & Li, M. (2017*b*). US Patent 20170123314.

[bb9] McKinnon, J. J., Jayatilaka, D. & Spackman, M. A. (2007). *Chem. Commun.* pp. 3814–3816. 10.1039/b704980c 18217656

[bb10] Noda, H. (2010). Jpn Patent 2010202714.

[bb11] Novozhilova, N. V., Magomedova, N. S., Sobolev, A. N. & Bel’skii, V. K. (1989*a*). *J. Struct. Chem.* **30**, 515–518.

[bb12] Novozhilova, N. V., Magomedova, N. S., Sobolev, A. N. & Bel’skii, V. K. (1989*b*). *J. Struct. Chem.* **30**, 635–639.

[bb13] Ogata, E., Yanase, N. & Kitahara, T. (2004). Jpn Patent 2004331527.

[bb14] Oster, T. A. & Coleman, M. T. (2011). WIPO Patent 2011049940.

[bb15] Rigaku OD (2023). *CrysAlis PRO*. Rigaku Oxford Diffraction Ltd, Yarnton, Oxfordshire, England.

[bb16] Sakamoto, S. & Iida, Y. (2014). Jpn Patent 2014193955.

[bb17] Sheldrick, G. M. (2015*a*). *Acta Cryst.* A**71**, 3–8.

[bb18] Sheldrick, G. M. (2015*b*). *Acta Cryst.* C**71**, 3–8.

[bb19] Spackman, M. A. & Jayatilaka, D. (2009). *CrystEngComm* **11**, 19–32.

[bb20] Spackman, P. R., Turner, M. J., McKinnon, J. J., Wolff, S. K., Grimwood, D. J., Jayatilaka, D. & Spackman, M. A. (2021). *J. Appl. Cryst.* **54**, 1006–1011. 10.1107/S1600576721002910 PMC8202033 34188619

[bb21] Terada, A. (2009). Jpn Patent 2009209219.

[bb22] Vouyiouka, S. N., Papaspyrides, C. D., Weber, J. N. & Marks, D. N. (2007). *Polymer* **48**, 4982–4989.

[bb23] Xiong, L.-K., Fu, Y.-F., Zhang, S.-Y. & Yin, C.-Y. (2016). *Fibers Polym.* **17**, 984–991.

